# Effect of Vitamin D-Enriched Gouda-Type Cheese Consumption on Biochemical Markers of Bone Metabolism in Postmenopausal Women in Greece

**DOI:** 10.3390/nu13092985

**Published:** 2021-08-27

**Authors:** George Moschonis, Ellen GHM van den Heuvel, Christina Mavrogianni, Yannis Manios

**Affiliations:** 1 Department of Dietetics, Nutrition and Sport, School of Allied Health, Human Services and Sport, La Trobe University, Melbourne 3086, Australia; 2 FrieslandCampina, Stationsplein 4, Post Box 1551, 3800 BN Amersfoort, The Netherlands; eghm.vandenheuvel@outlook.com; 3 Department of Nutrition and Dietetics, School of Health Science and Education, Harokopio University, 17671 Athens, Greece; cmavrog@hua.gr (C.M.); manios@hua.gr (Y.M.)

**Keywords:** postmenopausal women, vitamin D, bone remodeling, cheese, enriched dairy

## Abstract

Considering the role of bone metabolism in understanding the pathogenesis of osteoporosis, the aim of the present study was to examine the effects of vitamin D-enriched cheese on the serum concentrations of the parathyroid hormone (PTH) and certain bone remodeling biomarkers in postmenopausal women in Greece. In a randomised, controlled dietary intervention, 79 postmenopausal women (55–75 years old) were randomly allocated either to a control (CG: n = 39) or an intervention group (IG: n = 40), consuming 60 g of either non-enriched or vitamin D3-enriched Gouda-type cheese (5.7 μg of vitamin D3), respectively, daily and for eight weeks during the winter. The serum concentrations of 25-hydroxy vitamin D (25(OH)D), PTH, bone formation (i.e., osteocalcin, P1NP) and bone resorption (i.e., TRAP-5b) biomarkers were measured. Consumption of the vitamin D-enriched cheese led to higher serum 25(OH)D concentrations of 23.4 ± 6.39 (*p* = 0.022) and 13.4 ± 1.35 (*p* < 0.001) nmol/L in vitamin D-insufficient women being at menopause for less and more than 5 years, respectively. In vitamin D-insufficient women that were less than 5 years at menopause, consumption of vitamin D-enriched cheese was also associated with lower serum PTH (Beta −0.63 ± 1.11; *p* < 0.001) and TRAP-5b (Beta −0.65 ± 0.23; *p* = 0.004) levels at follow-up, compared with the CG. The present study showed that daily intake of 5.7 μg of vitamin D through enriched cheese increased serum 25(OH)D concentrations, prevented PTH increase and reduced bone resorption in vitamin D-insufficient early postmenopausal women, thus reflecting a potential food-based solution for reducing the risk of bone loss occurring after menopause.

## 1. Introduction

In 2010, the US Institute of Medicine (IOM) released the latest and most updated Dietary Reference Intake (DRI) for vitamin D, which was mainly based on evidence indicating the beneficial effects of vitamin D on musculoskeletal health outcomes, such as fractures, osteomalacia, rickets, muscle weakness, falls, etc. [1]. The DRIs of vitamin D have been also established based on the effects of dietary vitamin D intake on serum 25-hydroxy vitamin D (25(OH)D) concentrations and the intention of keeping them at levels equal or above 50 nmol/L, which is the diagnostic threshold for vitamin D sufficiency [2]. This is because serum concentrations of 25(OH)D of at least 50 nmol/L are the basic requirement for the normalisation of parathyroid hormone (PTH) levels in blood [3]. As both vitamin D and PTH are involved in controlling calcium and bone metabolism, the clinical outcomes of vitamin D insufficiency (i.e., 25(OH)D < 50 nmol/L) and the consequent secondary hyperparathyroidism in the skeleton is an accelerated rate of bone remodeling, which if sustained gradually leads to bone loss [3]. Bone remodeling describes a dynamic process that is reflected in the interrelated function of bone-forming osteoblasts and bone-resorbing osteoclasts [4,5]. During bone resorption, osteoclastic activity leads to the release of breakdown products of type-I collagen, a process that is usually mediated by certain enzymes, such as the osteoclast-specific 5b isoform of tartrate-resistant acid phosphatase (TRAP-5b) [6]. In healthy bone, the resorption cavity created by osteoclasts is filled with new osteoid material secreted by active osteoblasts. As part of this process, one of the bone formation molecules secreted by active osteoblasts is osteocalcin (OC), which binds to the mineralised bone matrix [7]. Another typical bone formation biomarker is type I procollagen-N-propeptide (P1NP), which is a byproduct of collagen synthesis. More specifically, following the synthesis of new type I collagen within the osteoblast, P1NP is cleaved from type I procollagen by proteases outside the osteoblast [6,8].

Women lose on average about 2% to 2.5% of their bone during the transmenopause period, which is one year before and two years after the last menstrual period [9]. Nevertheless, the rapid decline in bone mineral density (BMD) occurs mainly after menopause, and as such, the cumulative 10-year lumbar spine BMD loss reported for Caucasian women is 10.6% [10], with most of it lost within the first 3 years after menopause, thus highlighting a time-period during which the risk of osteoporotic fractures is elevated [11]. Besides menopause in women, some of the main risk factors for the progression and development of bone loss are also related to lifestyle and particularly to inadequate dietary intake of certain essential micronutrients [12]. The combined intake of both vitamin D and calcium results in a significant reduction in non-vertebral fractures, especially in those individuals with insufficient vitamin D status [13,14]. Even though there is an abundance of clinical trials indicating the beneficial effects of calcium and/or vitamin D supplementation on the prevention of bone loss and on the levels of bone remodeling biomarkers [15,16,17,18,19], there are extremely few dietary intervention studies examining the effect of increased intake of these micronutrients through fortified foods [20]. Previously, we showed that consumption of 60 g of reduced-fat Gouda-type cheese enriched with vitamin D3 was effective in decreasing the prevalence of vitamin D insufficiency during winter months in a population of postmenopausal women in Greece [21].

Considering the emerging role of bone metabolism in understanding the pathogenesis of osteoporosis, the primary aim of the present study was to examine the effects of vitamin D-enriched cheese on the serum concentrations of PTH and certain bone remodelling biomarkers in early or late postmenopausal women in Greece, with sufficient or insufficient vitamin D status at the start of the intervention.

## 2. Materials and Methods

### 2.1. Study Design and Sampling Procedures

The current study was a single-blinded (i.e., blinded only to study participants), randomised controlled dietary intervention study, testing the effect of vitamin D_3_-enriched, reduced-fat Gouda-type cheese on the serum concentrations of certain calciotropic hormones (i.e., 25(OH)D, PTH), bone formation (i.e., OC, P1NP) and bone resorption markers (i.e., TRAP-5b) in postmenopausal women.

The sampling procedures followed in the present study, which was originally designed to assess vitamin D status changes as a primary outcome, have been described in detail elsewhere [21]. According to sample size estimation, a sample of 37 women per treatment was adequate to provide statistical power greater than 80% (alpha = 0.05, two-tailed) for detecting an increase in serum 25(OH)D levels of ~6 nmol/L from baseline to follow-up at probability of type I error <0.05. To account for potential dropouts, the number of women was increased to 40 per group.

### 2.2. Screening and Ethics Approval

The study was initiated with two screening phases in October 2014, when volunteers were invited to participate via informational brochures and posters which were distributed in public buildings and community centres in two municipalities within the wider district of Athens, namely Kallithea and Tavros-Moschato. Women eligible to participate in the study were those that had no disease/pathology that interacts with vitamin D metabolism; those not requiring or taking any medications, (including hormone replacement therapy) that interact with vitamin D metabolism or vitamin D supplements for medical reasons (e.g., osteoporosis); those not having cow’s milk allergy or a history of drug and/or alcohol abuse; those who used to consume cheese daily; those that had not a planned vacation to a sunny holiday destination during the intervention period; and those who were more than 5 years post-menopause. However, the screening process resulted in a small number of women that were less than 5 years since menopause, but as they were close to the 5-year threshold and were satisfying all other inclusion criteria they were also included in the study. Through the first screening phase, 135 women (aged 55–75 y) satisfying the inclusion criteria were identified and were invited to participate in the second screening (December 2014).

The second screening was carried out via scheduled meetings and personal sessions with each one of the 135 eligible women at the Metabolic Unit of the Laboratory of Nutrition and Clinical Dietetics (LNCD) at Harokopio University. As described in detail previously [21], this second screening phase yielded 80 eligible women having, for the most part, homogenous characteristics at baseline. All study participants signed written informed consent forms. The study was approved by the Ethics Committee of Harokopio University (Approval code: 43/23-07-2014) and was conducted in accordance with the code of ethics of the World Medical Association (Declaration of Helsinki) for experiments involving humans. The study protocol registration number was ClinicalTrials.gov: NCT02543671.

### 2.3. Treatments and Intervention

The 80 eligible women identified after the second screening phase were randomly allocated to an intervention group (IG) and a control group (CG) using a random sampling approach [21]. Four weeks prior to the initiation of the intervention, any eligible participants reporting the use of Vitamin D dietary supplements were instructed to discontinue any intake of them (i.e., one woman in the CG and one woman in the IG). This washout period was included prior to the initiation of the intervention, in order to avoid or minimise any possible effect of supplement use on 25(OH)D concentrations at baseline. Study participants were provided with and were also instructed to consume (as part of their usual diet), 60 g of non-enriched reduced-fat Gouda-type cheese (CG; n = 40) or vitamin D3-enriched, reduced-fat Gouda-type cheese (IG; n = 40) for eight weeks during the winter (i.e., from January to March 2015). Both types of Gouda cheese (i.e., the vitamin D-enriched and the non-enriched type) examined in the present study were made from cow’s milk and provided the same amount of several important nutrients, such as protein, phosphorus, calcium, zinc and vitamin B12. However, the 60 g of the vitamin D3-enriched, reduced-fat Gouda-type cheese also provided 5.7 μg of vitamin D per day, which was independently verified using a liquid chromatography–mass spectrometry-based method, as previously described for pork meat [22].

All study participants were instructed to equally replace their habitual consumption of cheese with the corresponding amount of the experimental cheese, so as to compensate for additional calories. In addition, they received detailed guidance by the research group members to report any signs of illness, use of medication and any deviations from the study protocol. Meetings with participants were held biweekly on campus at Harokopio University in Athens, to supply to them the amount of cheese required for the next two weeks. Compliance to the intervention scheme (i.e., adherence to daily consumption of the 60 g of experimental cheese) was also assessed, as described in detail elsewhere [21].

### 2.4. Measurements

The effectiveness of the intervention was assessed with the assessment of dietary intake and clinical examinations conducted at baseline and follow-up using the same procedures and methodology. The dietary data obtained at the second screening of the study were used as baseline intake data. The follow-up examination took place in mid-March 2015, after eight weeks of intervention.

#### 2.4.1. Dietary Intake

An existing quantitative FFQ validated for calcium intake [23] was updated to also include vitamin D dietary sources (such as fish/seafood, beef, pork, mushrooms, eggs and margarine) and was used to assess habitual dietary calcium and vitamin D intakes. The mean content of the foods listed in the FFQ in calcium and vitamin D was multiplied to their daily frequency of consumption, thus providing the amount of habitual, mean daily calcium and vitamin D intakes.

#### 2.4.2. Anthropometry

Body weight and standing height were measured following the procedures that have been previously described in detail [21]. Body mass index (BMI) (in kg/m^2^) was calculated by dividing body weight to standing height squared.

#### 2.4.3. Blood Analyses

Early morning venous blood samples were obtained from each study participant following a 12 h overnight fast at baseline and follow-up examination. Blood was collected in vacutainers without added anticoagulant and was kept at room temperature for ≈2 h, where it was allowed to clot. Centrifugation for serum separation was conducted at 3000 rpm for 15 min. Aliquots of 1.5 mL serum were then stored at −80 °C. Serum concentrations of total 25(OH)D (i.e., 25(OH)D_2_ plus 25(OH)D_3_) in all serum samples were measured in the Cork Centre for Vitamin D and Nutrition at University College Cork, using a liquid chromatography–tandem mass spectrometry method, described in detail elsewhere [21,24]. Based on the obtained measurements, vitamin D insufficiency and deficiency were defined at serum 25(OH)D concentrations below 50 and 30 nmol/L, respectively [25].

Serum intact PTH and P1NP concentrations were measured with electrochemiluminescence immunoassay (ECLIA) (Roche Diagnostics, Mannheim, Germany) using an ECLIA Elecsys autoanalyser (Cobas e 601). Furthermore, serum total OC concentrations were measured using chemiluminescence immunoassay (CLIA) (Immulite 2000xpi Siemens Healthcare, Erlagen, Germany). In addition, the concentrations of TRAP-5b were measured using an immunocapture enzymatic assay (MetraTRAP-5b EIA Kit, Quidel Corporation, San Diego, CA, USA). The intra-assay and inter-assay coefficients of variation of the biochemical assays performed to measure the concentrations of the aforementioned biochemical indices were: less than 6% for PTH, less than 5% for P1NP; 2.8% and 4.5%, respectively, for osteocalcin; and less than 3% for TRAP-5b.

#### 2.4.4. Statistical Analysis

Descriptive data were reported as mean and standard deviations (SD) for continuous variables or as frequencies (n) and percentages (%) for categorical ones. The Kolmogorov–Smirnov test was used to determine normality of distribution of the examined continuous variables. Between-group differences at baseline were assessed with Student’s *t*-test. The chi-squared test was also used to examine between-group differences in the percentages of subjects with serum 25(OH)D levels below 30 and 50 nmol/L at baseline. General estimating equations (GEE) were applied with correction for baseline values in order to examine the effect of treatment with the vitamin D-enriched cheese versus the control group (independent variable) on the serum concentrations of calciotropic hormones and bone remodelling biomarkers at follow-up (dependent variables). Interaction was studied by adding a product term of group (i.e., intervention vs. control group) times vitamin D status (i.e., 25(OH)D < 50 nmol/L vs. 25(OH)D ≥ 50 nmol/L) or years since menopause (i.e., ≤ vs. >5 years). A *p*-value lower than 0.1 for the interaction term was considered significant. The analysis was stratified by insufficiency or sufficiency in participants’ vitamin D status (i.e., 25(OH)D< or ≥50 nmol/L, respectively) and by years since menopause. Subsequently, within each stratum, the other potential effect modifiers were tested. Years since menopause (i.e., ≤ or >5 years) remained significant for PTH and TRAP-5b, and cases of vitamin D sufficiency were also significant for OC. To obtain the regression coefficient for those women that were at menopause for more than 5 years, the coding of the categorial variable was reversed (0 = 1, and 1 = 0). The cut-off of 5 years since menopause was chosen as after this time threshold, the hormone levels in women are more balanced [26]. All models were adjusted for potential confounding factors that changed the regression coefficients of the treatment by 10% or more when added to the linear regression model. The SPSS statistical analysis software for Windows, version 24.0 (SPSS Inc., Chicago, IL, USA) was used to perform all statistical analyses. All statistical tests were two-tailed, and the level of statistical significance was set at *p* < 0.05.

## 3. Results

### 3.1. Baseline Characteristics of Study Participants

**Figure 1:**
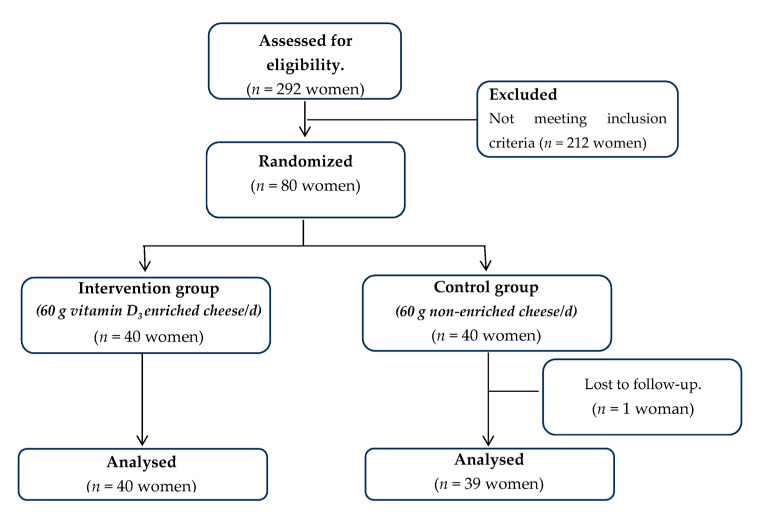
CONSORT flow diagram of study participants.

From the 80 eligible women that were randomly allocated to the two treatments, 79 (i.e., 39 in the control group and 40 in the intervention group) completed the study having full data at both baseline and follow-up examination (Figure 1).

Table 1 presents the mean sociodemographic characteristics (i.e., age, years since menopause and years of education) of study participants by treatment at baseline, as well as their BMI, serum concentrations of calciotropic hormones (i.e., 25(OH)D and PTH), bone formation (i.e., OC and P1NP) and bone resorption (i.e., TRAP-5b) markers. In addition, Table 1 presents the prevalence of vitamin D deficiency (25(OH)D < 30 nmol/L) and insufficiency (25(OH)D < 50 nmol/L) by treatment arm. No statistically significant differences were observed between the two treatment arms in any of the aforementioned characteristics and blood biomarkers.

### 3.2. Intervention Effect on Serum 25(OH)D Levels in the Total Sample and Vitamin D-Insufficient Women

As described in detail elsewhere [21], cheese consumption resulted in significant increases in dietary calcium intake in both treatment arms, by 191 mg/day (95% CI 103–279) in the IG and 157 mg/day (95% CI 6.7–247) in the CG over the eight weeks, but there was no difference between groups (*p* = 0.6). Similarly, dietary protein intake was equally increased in both treatment arms, since the addition of cheese was the only change introduced in the dietary intake of study participants. Considering that both treatments consumed 60 g of Gouda cheese, protein intake in both the intervention and the control group increased by 18.2 g/day. Mean dietary vitamin D intake increased significantly from baseline to follow-up only in the IG, while it decreased significantly in the CG over the same period, and these changes were also found to be significantly different between groups (5.1 μg (95% CI 4.7–5.5) vs. −0.6 μg (−1.1 to −0.2) *p* < 0.001, respectively). Further to these dietary intake changes, mean serum 25(OH)D concentration increased by 5.1 nmol/L in the IG and decreased by 4.6 nmol/L in the CG during the 8-week intervention period (*p* < 0.001). In addition, 31 out of 40 women in the IG (i.e., 77.5%) responded positively to the implemented intervention by increasing their serum 25(OH)D concentrations by at least 1.08 nmol/L. The increase in serum 25(OH)D concentrations was more pronounced in the vitamin D-insufficient women in the IG, since positive changes were observed for 25 out of 27 (i.e., 92.6%) women consuming the vitamin D-enriched cheese.

### 3.3. Effect of Vitamin D-Enriched Cheese Consumption on Bone Metabolism Biomarkers

Table 2 presents the GEE regression coefficients by vitamin D status of the under-study population of postmenopausal women. Women in the IG that consumed the vitamin D-enriched cheese and were vitamin D-insufficient women (25(OH)D < 50 nmol/L) at baseline were found to have higher 25(OH)D levels (Beta 13.8 ± 1.28; *p* < 0.001) and lower levels of the bone resorption marker TRAP-5b (Beta −0.20 ± 0.12; *p* = 0.050) at follow-up, compared with the CG that consumed the non-enriched cheese. Furthermore, women in the IG that were vitamin D-sufficient (25(OH)D ≥ 50 nmol/L) at baseline were found to have significantly higher levels of the bone formation marker P1NP (Beta 6.12 ± 2.42; *p* = 0.011) compared with women in the CG. No other significant between-group differences were observed, either in vitamin D-insufficient or in vitamin D-sufficient women.

Table 3 shows the GEE regression coefficients by vitamin D status and years since menopause. In women that were vitamin D-insufficient at baseline and were in menopause for less than 5 years, consumption of vitamin D-enriched cheese was found to be associated with higher 25(OH)D levels (Beta 23.4 ± 6.39; *p* = 0.022) and lower levels of serum PTH (Beta −0.63 ± 1.11; *p* < 0.001) and TRAP-5b (Beta −0.65 ± 0.23; *p* = 0.004) at follow-up, compared with the CG that consumed the non-enriched cheese. Women that were vitamin D-insufficient at baseline and for more than 5 years in menopause were also found to have higher concentrations of 25(OH)D (Beta 13.4 ± 1.35; *p* < 0.001) at follow-up, compared with the CG, but there were no other significant associations observed with serum PTH and bone resorption markers. In women that were vitamin D-sufficient at baseline and less than 5 years in menopause, consumption of the vitamin D-enriched cheese was positively associated with the serum concentrations of osteocalcin (Beta 12.6 ± 1.46; *p* < 0.001), P1NP (Beta 6.24 ± 2.35; *p* = 0.008) and TRAP-5b (Beta 0.39 ± 0.11; *p* < 0.001). A positive association was also observed with P1NP concentrations (Beta 6.24 ± 2.35; *p* = 0.008) in vitamin D-sufficient women with more than 5 years since their menopause.

## 4. Discussion

The present single-blinded, randomised, controlled trial examined the effect of vitamin D-enriched cheese consumption that provided 5.7 mcg of vitamin D_3_ per day on the serum concentrations of certain calciotropic hormones and bone remodeling biomarkers on postmenopausal women living in Greece. The intervention was implemented from January to March, which is a typical winter period in Greece, and resulted in significant drops of serum 25(OH)D levels in the wider population due to limited sunlight exposure [27,28]. As previously reported [21], consumption of the vitamin D_3_-enriched Gouda-type cheese significantly increased serum 25(OH)D concentrations by an average of 6.4 nmol/L from baseline in the examined postmenopausal women.

The present study is adding to these previous findings, reporting significant effects of the implemented vitamin D treatment on the examined biomarkers of bone metabolism and in women with different vitamin D and menopausal status. In this regard, in women that were vitamin D-insufficient at baseline and were allocated to the treatment arm that consumed the vitamin D-enriched cheese, 25(OH)D levels at follow-up were higher by an average of 23.4 and 13.4 nmol/L in those being at menopause for less and more than 5 years, respectively, compared with those consuming the non-enriched cheese. The increases reported by the present study in 25(OH)D levels are much higher than those reported by other similar studies on vitamin D-insufficient women. Bonjour et al. [29] observed a significant increase in serum 25(OH)D concentration of 9 nmol/L in postmenopausal women (mean age 57.1 ± 3.9 years), which were vitamin D-insufficient at study entry, following consumption of vitamin D-enriched cheese providing a daily dose of 2.5 mcg of vitamin D. The much higher 25(OH)D concentrations observed in the present study in early postmenopausal women (i.e., those being ≤5 years at menopause) consuming the vitamin D-enriched cheese, may also explain the lower PTH levels that were observed in the IG at follow-up, compared with the CG. These findings reflect the well-documented inverse relationship between 25(OH)D and PTH levels [30,31] and indicate that even a moderate supplemented daily dose of 5.7 mcg of vitamin D is adequate to inhibit the increase in PTH in blood among vitamin D-insufficient individuals.

The lowering effect of treatment with the vitamin D-enriched cheese on the serum PTH levels of the vitamin D-insufficient early postmenopausal women, could have also resulted to a lower rate of bone resorption, as evidenced by the lower levels of TRAP-5b at follow-up observed in the IG compared with their counterparts in the CG that consumed the non-enriched cheese. Other dietary intervention trials examining the effects of vitamin D-enriched dairy products on biomarkers of bone metabolism have also reported a suppression of bone resorption, mainly through a decrease in TRAP-5b concentrations [5,29,32,33]. TRAP-5b seems to have an advantage against other bone resorption biomarkers, since it is a lysosomal enzyme specific only to osteoclast activity [4], having a much lower day-by-day and within-subject variability as compared with urinary and other serum telopeptide molecules [34]. The above is of particular importance, particularly in trials with smaller sample sizes and thereby lower statistical power, as with the present study. In addition, TRAP-5b has been also reported to have a higher specificity and sensitivity in comparison with other bone resorption biomarkers [35], although it should be noted that CTX-1 is the recommended bone resorption biomarker by the International Osteoporosis Foundation and the International Federation of Clinical Chemistry and Laboratory Medicine [36]. Regarding vitamin D-insufficient women that were at menopause for more than 5 years, although the average serum concentration of 25(OH)D at follow-up was higher in the study group that consumed the vitamin D-enriched cheese, there was no significant effect observed on either PTH or bone resorption biomarkers markers. This probably indicates that the need of an intervention is more pronounced in vitamin D-insufficient women that are in their early postmenopausal phase, for whom the rate of bone turnover is accelerated [37].

In vitamin D-sufficient women, consumption of the vitamin D-enriched cheese had no effect on their vitamin D status, as there were no differences observed in 25(OH)D levels at follow-up between women allocated to the two treatment arms. However, as indicated by the significant positive beta coefficients reported in Table 2, early postmenopausal women (i.e., <5 years since menopause) with sufficient vitamin D levels (i.e., 25(OH)D > 50 nmol/L) in the IG were found to have higher serum levels of both bone formation (i.e., osteocalcin and P1NP) and bone resorption (i.e., TRAP-5b) markers compared with the CG, thus indicating an increased level of bone turnover. Increased rate of bone turnover has been associated with bone loss and fracture risk, particularly in individuals for whom vitamin D insufficiency coexists with hyperparathyroidism and low dietary calcium intake [38,39]. However, in the present study the increases observed in the levels of bone formation and bone resorption markers did not coincide with any of the above, since women that consumed the vitamin D-enriched cheese had sufficient levels of 25(OH)D and did not show any increase in their PTH levels, while their dietary calcium intake remained adequate throughout the study at levels higher than 900 mg per day [21]. In this regard, the observed acceleration in the rate of bone turnover could be attributed to other aetiological factors, with oestrogen deficiency occurring after menopause being one of them. It is known that the halt in oestrogen occurring after menopause in women is associated with increased bone turnover [40], which leads to a 9–10% decrease in bone mineral density (BMD), especially during the first 3 to 5 years after menopause [10]. In this regard, the lack of oestrogens might also negatively influence vitamin D-regulated processes, such as intestinal calcium absorption [41]. The above implies that the bone-enhancing effect of vitamin D-enriched cheese during menopause transition requires further study.

Cheese is an extremely good food source of both calcium and protein. In the present study, the addition of cheese in participants’ diets increased both protein and calcium intake in both treatment arms, while those consuming the enriched cheese also increased their vitamin D intake. Based on the current knowledge, there is a synergy between vitamin D and calcium in terms of reducing the rate of bone resorption and turnover, at least in women with a low intake of calcium or vitamin D status [42]. Furthermore, high dietary protein intake stimulates the production of the anabolic hormone, insulin-like growth factor-I (IGF-I), which is important in bone formation, and has also been shown to have a mediating role in vitamin D metabolism. In this regard, higher circulating IGF-I levels have been reported to enhance renal production of 1,25-dihydroxyvitamin D, which also stimulates bone formation [42]. The synergy between dietary protein intake and vitamin D was also confirmed in a retrospective analysis with the use of data from a 3-year RCT examining calcium and vitamin D supplementation on 342 healthy people (≥65 years old) [43]. Those participants who completed the trial and were categorised in the highest tertile of dietary protein intake had an additional synergistic effect of higher protein, vitamin D and calcium intake on BMD at the femoral neck and total body [43]. In the present study, the interaction between dietary protein, calcium and higher baseline serum 25(OH)D concentrations in vitamin D-sufficient women may explain the increase in bone formation due to the vitamin D-enriched cheese in the vitamin D-sufficient women, while not showing an increase in 25(OH)D. In vitamin D-sufficient women who were still in menopause transition, oestrogen levels might have interfered with treatment, leading to an increase in both bone formation and bone resorption. In this context, the final effect of vitamin D-enriched cheese consumption on bone metabolism, and subsequently on BMD in vitamin D-sufficient women, will ultimately depend on the interactions between protein and calcium intake, vitamin D and PTH levels, as well as the decrease in oestrogen levels occurring after menopause. However, there is still a need for further study to confirm all aforementioned interactions on postmenopausal women’s bone metabolism.

The findings of the present study should be interpreted in light of its strengths and limitations. The main strengths of the study are its randomised placebo-controlled design and the focus on vitamin D-insufficient women, who are most in need of a targeted intervention with vitamin D supplementation. Other important strengths include the detailed study protocols and procedures, which were tightly followed to assure the correct implementation of the intervention, the independent verification of the vitamin D content of foods and the high adherence of participants to treatment, as also confirmed by the procedures followed to record cheese consumption. Limitations of the present study are the relatively small sample size, especially of women that were up to 5 years post-menopause. In addition, although the study was adequately powered for the examination of the changes in its primary outcome, this might not be the case for the secondary outcomes examined in the present study, i.e., the serum levels of PTH and bone remodeling biomarkers. Another limitation of the current study was also its single-blinded design (i.e., blinded only to study participants). However, although those research team members that distributed the two different types of cheese to study participants were not blinded to treatment, the researchers undertaking, and subsequently reporting, the biochemical outcome measures were masked to all participants’ allocation scheme. Furthermore, the 2 h waiting period might have been too long and may have resulted in the degradation of some sensitive biomarkers, such as osteocalcin. However, based on the standardised protocol followed in the clinical chemistry laboratory, where all biochemical analyses took place, the 2 h waiting period was an acceptable time for allowing the blood to adequately clot and avoiding haemolysis during centrifugation, while it only had minimal or no effect on most of the examined bone remodeling biomarkers. Lastly, although the biological variability of the examined bone remodelling biomarkers might have a confounding effect on the study findings, the methods followed in the present study for collecting, processing and analysing blood to determine the concentrations of bone remodeling biomarkers in serum were standardised, and were the same for all study participants and in both time points of data collection. In this regard, any biological variability produced a systematic error that was equally affecting subjects in both the intervention and the control group.

## 5. Conclusions

In conclusion, the present dietary intervention with Gouda-type cheese enriched with vitamin D significantly increased serum 25(OH)D concentrations, prevented PTH increase and reduced bone resorption in vitamin D-insufficient early postmenopausal women. In vitamin D-sufficient women, an interaction between the extra protein and calcium from the cheese combined with the higher vitamin D status might explain the increase in the concentration of bone formation markers. This increase in bone formation seemed to coincide with an increase in bone resorption and consequently turnover in women that were less than 5 years at menopause, which might be due to interference with declining oestrogen levels. As reduction in bone resorption observed in vitamin D-insufficient women concurs with increased 25(OH)D concentrations, the findings of the present study could reflect a novel food-based approach in improving vitamin D status in this susceptible population group and in producing some favourable changes on bone metabolism that may be protective against bone loss that occurs post-menopause. However, the interaction between the consumption of vitamin D-enriched cheese with serum 25(OH)D concentrations and oestrogen insufficiency in vitamin D-sufficient postmenopausal women requires further investigation.

## Figures and Tables

**Table 1:** Baseline descriptive characteristics of study participants completing the trial presented by treatment.

	Intervention Group (n = 40)	Control Group (n = 39)	*p*-Value *
**Sociodemographic indices**	**Mean (SD)**		
Age (years)	62.6 (6.0)	63.2 (5.9)	0.670
Years since Menopause	13.9 (5.7)	14.2 (7.3)	0.805
Education (years)	12.3 (3.8)	12.7 (3.5)	0.594
**Anthropometric indices**			
Body Mass Index (kg/m^2^)	28.0 (3.8)	29.0 (2.9)	0.190
**Calciotropic hormones**			
25(OH)D (nmol/L)	47.3 (15.2)	42.9 (17.7)	0.105
PTH (pg/mL)	41.9 (10.6)	44.2 (13.9)	0.415
**Bone formation markers**			
Osteocalcin (ng/mL)	24.0 (8.26)	23.6 (7.12)	0.819
P1NP (ng/mL)	50.5 (12.2)	52.5 (14.4)	0.522
**Bone resorption markers**			
TRAP-5b (U/L)	2.52 (1.03)	2.39 (0.76)	0.533
**Vitamin D deficiency**	**n (%)**		
Serum 25(OH)D < 30 nmol/L	5 (12.5)	10 (25.0)	0.137 ^‡^
**Vitamin D insufficiency**			
Serum 25(OH)D < 50 nmol/L	27 (67.5)	24 (62.5)	0.750 ^‡^

* *p*-values were derived from Student’s *t*-test, unless stated otherwise. ^‡^ *p*-values were derived from the chi-squared test.

**Table 2:** Effect of the intervention with vitamin D-enriched cheese compared with the control group (independent variable) on serum concentrations of calciotropic hormones and bone resorption markers at follow-up (dependent variables), by vitamin D status at baseline.

	Vitamin D Status					
	25(OH)D < 50 nmol/L (n = 48)			25(OH)D ≥ 50 nmol/L (n = 31)		
Dependent Variables	Beta	SE	*p*-Value	Beta	SE	*p*-Value
*Calciotropic hormones*						
25(OH)D (nmol/L)	13.8	1.28	<0.001	4.48	2.01	0.126
PTH (pg/mL)	−0.91	0.95	0.067	1.15	1.09	0.108
*Bone formation markers*						
Osteocalcin (ng/mL)	0.12	0.81	0.883	1.54	1.11	0.166
P1NP (ng/mL)	−0.26	2.13	0.904	6.12	2.42	0.011
*Bone resorption markers*						
TRAP-5b (U/L)	−0.20	0.12	0.050	0.12	0.13	0.348

*p*-values obtained by GEE corrected for baseline values and because of significant interactions shown per group.

**Table 3:** Effect of the intervention with vitamin D-enriched cheese compared with the control group (independent variable) on serum concentrations of calciotropic hormones and bone resorption markers at follow-up (dependent variables), by vitamin D status at baseline and years since menopause.

	Vitamin D Status											
	25(OH)D < 50 nmol/L						25(OH)D ≥ 50 nmol/L					
	Years since Menopause						Years since Menopause					
	≤5 Years (n = 6)			>5 Years (n = 42)			≤5 Years (n = 3)			>5 Years (n = 28)		
Dependent Variables	Beta	SE	*p*-Value	Beta	SE	*p*-Value	Beta	SE	*p*-Value	Beta	SE	*p*-Value
*Calciotropic hormones*												
25(OH)D (nmol/L)	23.4	6.39	0.022	13.4	1.35	<0.001	6.50	11.4	0.669	3.14	2.28	0.182
PTH (pg/mL)	−0.63	1.11	<0.001	−0.95	1.05	0.301	−0.86	1.11	0.165	1.16	1.10	0.110
*Bone formation markers*												
Osteocalcin (ng/mL)	0.04	0.81	0.959	0.04	0.81	0.959	12.61	1.46	<0.001	0.55	1.04	0.598
P1NP (ng/mL)	−0.05	2.03	0.981	−0.05	2.03	0.981	6.24	2.35	0.008	6.24	2.35	0.008
*Bone resorption markers*												
TRAP-5b (U/L)	−0.65	0.23	0.004	−0.15	0.11	0.158	0.39	0.11	<0.001	0.04	0.13	0.750

*p*-values obtained by GEE corrected for baseline values and because of significant interactions shown per group.
